# A Diagnosis of Biophysical and Socio-Economic Factors Influencing Farmers’ Choice to Adopt Organic or Conventional Farming Systems for Cotton Production

**DOI:** 10.3389/fpls.2017.01289

**Published:** 2017-07-19

**Authors:** Amritbir Riar, Lokendra S. Mandloi, Randhir S. Poswal, Monika M. Messmer, Gurbir S. Bhullar

**Affiliations:** ^1^Department of International Cooperation, Research Institute of Organic Agriculture Frick, Switzerland; ^2^bioRe Association India Khargone, India; ^3^Central Soil Salinity Research Institute Karnal, India; ^4^Agricultural Extension Division, Indian Council of Agricultural Research New Delhi, India; ^5^Department of Crop Science, Research Institute of Organic Agriculture Frick, Switzerland

**Keywords:** organic cotton, motivational factors, biophysical factors, socio-economic factors

## Abstract

Organic agriculture is one of the most widely known alternative production systems advocated for its benefits to soil, environment, health and economic well-being of farming communities. Rapid increase in the market demand for organic products presents a remarkable opportunity for expansion of organic agriculture. A thorough understanding of the context specific motivations of farmers for adoption of organic farming systems is important so that appropriate policy measures are put in place. With an aim of understanding the social and biophysical motivations of organic and conventional cotton farmers for following their respective farming practices, a detailed farm survey was conducted in Nimar valley of Madhya Pradesh state in central India. The study area was chosen for being an important region for cotton production, where established organic and conventional farms operate under comparable circumstances. We found considerable variation among organic and conventional farmers for their social and biophysical motivations. Organic farmers were motivated by the sustainability of cotton production and growing safer food without pesticides, whereas conventional farmers were sensitive about their reputation in community. Organic farmers with larger holdings were more concerned about closed nutrient cycles and reducing their dependence on external inputs, whereas medium and small holding organic farmers were clearly motivated by the premium price of organic cotton. Higher productivity was the only important motivation for conventional farmers with larger land holdings. We also found considerable yield gaps among different farms, both under conventional and organic management, that need to be addressed through extension and training. Our findings suggest that research and policy measures need to be directed toward strengthening of extension services, local capacity building, enhancing availability of suitable inputs and market access for organic farmers.

## Introduction

Global population is projected to reach 11 billion by the end of 21st century (Alexandratos and Bruinsma, 2012) further escalating the multifaceted challenges ahead of modern agricultural systems. In a scenario where nearly 1 billion people are currently undernourished (FAO, 2011) our agricultural system needs to ensure the provision of sufficient and affordable nutrition for everyone. About a billion hectares of additional cropland are needed by 2050 to meet the projected increase of 70–110% in global food demand using contemporary farming practices (Tilman et al., 2001; Bruinsma, 2009). Most of the additional land will come from developing countries mainly in the tropics, which will inhabit more than half of the world’s population by the middle of this century (Edelman et al., 2014). This makes further intensification of agro-ecosystems imminent, which needs to be brought in such a way that ecological balance of our planet is maintained (Andres and Bhullar, 2016).

In recent decades, fossil based input-intensive industrial agricultural technologies have been widely recognized as being unsustainable over the long-run (Pingali, 2012). Moreover, global food system has increasingly faced the impacts of escalating intensity of climatic extremes (Nelson et al., 2009) as well as economic uncertainties (Kastner et al., 2012). This has led to stronger calls for transformation of our agricultural system using holistic farming practices based on ecological principles. Several different alternative farming approaches have been put forward in different parts of the world from time to time with varying degree of success. Organic agriculture is one of the most widely known alternative agricultural production systems advocated for its benefits to soil, environment, health and economic condition of farming communities (Mäder et al., 2002; Badgley et al., 2007; Forster et al., 2013).

Steep growth in organic markets has resulted in global demand for organic products surpassing the total production (Sahota, 2016; Willer and Lernoud, 2016). Until recently, organic market was primarily dominated by the developed countries, where prosperous consumers can afford to pay premium prices for organic products. Organic sector in the developing countries has largely been export oriented, however, with rapid economic development domestic organic markets are currently seeing significant expansion in the emerging economies (Sirieix et al., 2011). There have been strong calls for mainstreaming of organic agriculture in some of the developing countries as well (Scialabba, 2000; UNCTAD-UNEP, 2008) and in some cases governments in different parts of the world have implemented pro-organic policies (Kolanu and Kumar, 2007; FAO, 2011; Wai, 2016). This presents a remarkable opportunity, particularly for small and medium holding farmers in developing countries (Rundgren, 2006). To fully utilize the available potential, appropriate implementation of policy measures is necessary, which demands a context specific understanding of available scenarios. For instance, the adoption rates of organic farming practices may vary among farmers depending upon various factors, including those of biophysical and socio-economic nature. Understanding the motivation of farmers for adoption of their specific set of agricultural management practices is of crucial importance to design suitable policy measures.

Since ancient times, India has been an important exporter of cotton. India regained its position as world’s largest producer of cotton in 2014–2015, as Indian farmers consistently produced over 6 million tons of cotton lint in 2013–2015. A dramatic change in the age old cotton cultivation practices in India happened in the second half of 20th century as the indigenous or ‘Desi’ varieties (*Gossypium arboreum*) were first replaced by American cotton (*Gossypium hirsutum*) varieties and hybrids and subsequently by genetically modified Bt-cotton. Because of the resistance to cotton bollworms and hence reduced pesticide usage, Bt-cotton was adopted by farmers relatively quickly after its first release in 2002 (Finger et al., 2011; James, 2011; Krishna and Qaim, 2012; Qaim and Kouser, 2013). Today, more than 95% of cotton produced in India is Bt-cotton, yet the impact of Bt-cotton adoption on farmers’ livelihood and environment is debated (Stone, 2011; Kathage and Qaim, 2012). Moreover, many reports of bollworms attaining resistance to Bt-toxin and emergence of secondary pests question the sustainability of this technology (Tabashnik, 1994; Luttrell et al., 2004; Bagla, 2010; Downes et al., 2010, 2016).

The productivity of cotton is limited by the following external factors: Scale of production, level of research support, local ginning capacity, access to quality seed, access to irrigation, access to timely inputs, production costs, price paid for seed cotton, access to credit, timely payment for the crop and availability of season-long farmer training (Page and Ritchie, 2009). The biggest sustainability challenge in conventional cotton production remains the need for high inputs of agrochemicals, many of which are known for their adverse effects on human health and potential harm to the environment (Page and Ritchie, 2009; Bachmann, 2012). Since most of the cotton produced in India is grown by smallholder, subsistence farmers usually with land holdings of less than one hectare, capital intensive high input farming is not the most suited choice for them. Organic production offers a suitable alternative to such farmers with potential advantages of lower expenses for farm inputs, healthier soils and environment as well as competitive gross margins (Rajendran et al., 2000; Lakhal et al., 2008; Forster et al., 2013). Despite the fact that only less than 5% of cotton produced in India is certified organic (Stone, 2011; Kathage and Qaim, 2012), India is still leading the global organic cotton production, as it contributed 66.9% of the worldwide production in 2014–2015 (Truscott et al., 2016). The global production of organic cotton saw a rapid growth from 2006 to 2010, which started to decline from 2011 onward (Truscott et al., 2016). With a steep increase in demand of organic fiber (Truscott et al., 2016), it is important to safeguard and increase the production of organic cotton in a sustainable manner.

Although India is a significant producer of organic crops, the bulk of organic production has been largely targeted at export markets. The share of domestic market is steadily increasing owing to the recent economic developments and consumer awareness (Chandrashekar, 2010). However, there is a strong need for development and implementation of appropriate policy measures considering the choices and motivations of farmers. This study was aimed at diagnosing the biophysical and socio-economic factors influencing the adoption of organic and conventional management practices by the cotton farmers in order to facilitate appropriate policy development. We hypothesized that the motivation of farmers for adoption of conventional or organic farming systems differs depending upon their awareness level, social perceptions, availability of resources and perceived profitability.

## Materials and Methods

### Study Region

This study was conducted in the Nimar valley of Madhya Pradesh state in central India, which is an agriculturally important region. In the study area, cotton is cultivated as a major cash crop, in rotation with other crops such as cereals, vegetables, and legumes (Myers and Stolton, 1999; Eyhorn et al., 2007). Studies comparing organic and conventional farming systems in this region have showed that performance of both the systems is somewhat comparable to each other (Eyhorn et al., 2007; Forster et al., 2013; Helfenstein et al., 2016). However, cotton yields in general are low and variable in Nimar valley and often do not reach the attainable levels on several farms of the region. This unique situation where conventional and contemporary organic agricultural systems are existing in parallel in a society with wide economic disparities offers a rigorous platform to understand the biophysical and socio-economic motivational characters of farmers. The main aim of this study was to identify social and biophysical motivational characters controlling rational decision of farmers to opt for either organic or conventional agricultural system at farm level.

### Farm Survey

During the cotton season of 2015 (May to December), a detailed structured survey of organic and conventional cotton farms was conducted in the cotton growing region of west Nimar. Survey questions were standardized in preliminary focussed group discussions with farmers, extension workers, research staff and other stakeholders using the joint innovation platform of the Research Institute of Organic Agriculture (FiBL) and bioRe Association (Andres et al., 2016). For structured survey, individual interviews were conducted at 60 organic and 60 conventional farms randomly selected from five different cotton growing pockets/clusters of west Nimar. Each farm was treated as a single operational unit and the farmer responsible for decision-making was interviewed. Farmers were selected solely based upon their farming practices, irrespective of farm size, soil type, education, income or any other demographic factors. In order to identify the social, biophysical and economic motivational factors behind adoption of a particular farming system (organic/conventional) by the farmers, the survey questionnaire included a section with a number of statements relating to views on farming practices and their sustainability. The farmers were asked to mark the category best describing their level of agreement with the statement (not, little, quite, very, and extremely). Additionally, survey respondents (farmers) had the possibility to add their own statements regarding major limiting factors for cotton production, in their preference to grow organic cotton and switching from conventional to organic. Upon careful consideration of each of such statements, they were grouped into thematically relevant categories.

For statistical analysis, farmers were further grouped according to size of their land holdings, in order to broadly represent different socio-economic categories. They were grouped into small (<2 ha), medium (2–4 ha), and large (>4 ha) holding farmers, with the small scale farmers recognized as being asset-poor (Singh et al., 2010; Coventry et al., 2015). Upon further subgrouping it was found that the number of respondents was too low in certain sub-categories to arrive at statistically sound conclusions per group. However, the number of respondents are sufficiently large to be able to discern issues and emerging trends. The survey targeted whole farm information on cotton crop management practices (including variety selection, fertilizer management, weed and pest management, number of picking) as well as the information on farmer demography and attitudes. Each farmer was personally visited by one of the designated staff members of bioRe extension team. The staff members were instructed in survey data compilation, to safeguard standardized survey data collection and preparation. Informed consent was obtained from all individual participants included in the study. The data were collected in an Excel document and to derive inferences Principal Component Analysis (PCA) were conducted on this data set.

### Principal Component Analysis

To do the PCA, the number of farmers selecting each of the limiting factors divided by the total number of farmers within each farm size group was calculated as a percentage using JMP (copyright SAS Institute Inc.) (Goupy and Creighton, 2007). Farming practices and farm size were included as factors and all the surveyed social and management related limiting factors were included as variables, and covariance was selected as the matrix type.

## Results and Discussion

### Profile of Respondents – Gender, Age, Education and Experience

Survey results show that farming is a means of livelihood in Nimar valley area and predominantly a male dominant profession as 86% of the total farm units surveyed were led by male farmers (**Table 1**). Interestingly, the proportion of farms managed by female farmers as operational head of farm was higher in organic farms group compared to conventional farms (17% v. 11%). Furthermore, the farm size showed a distinguishing feature, since the proportion of farms managed by female farmers was higher on farms with large land holdings both on conventional and organic farms (**Table 1**). This result is of particular significance since women are believed to be the quiet drivers of change toward more sustainable production systems and healthier diets (Altieri and Koohafkan, 2008). Women comprise of more than 40% of the agricultural labor force in developing countries and up to 50% in Asia and sub-Saharan Africa. In recent decades, development agencies and policy advocates have been emphasizing that women could increase the farm productivity by 20–30%, if they have the same access to productive resources as men (Lastarria-Cornhiel, 2006; Altieri and Koohafkan, 2008). However, in an extensive review of available literature, Doss (2015) found men and women to be equally productive, given the access to similar resources. In our study, the productivity of organic farms operated by females was statistically similar, i.e., 1410 ± 161 kg ha^-1^ and 1396 ± 121 kg ha^-1^ of organic farms operated by female and male farmers, respectively. Similar to organic farms, productivity of conventional farms led by male (1819 ± 123 kg ha^-1^) and female (1792 ± 327 kg ha^-1^) farmers also did not differ. Like all other faces of life, the participation of women at decision-making capacities has also increased in agriculture also in developed countries. According to a report by the US Department of Agriculture’s Economic Research Service, farms operated by women increased to 14% in 2007, up from 5% in 1978 (Hoppe and Korb, 2013). Some studies in developed countries (mainly Europe) have tried to generalize the differences among organic and conventional farmers based on their age, education, farming experience and land holding. For instance, Rigby and Cáceres (2001) characterized organic farmers in United Kingdom (UK) as typically smaller in terms of land holding with better education and of younger age with urban background and little experience. In our study, the organic and conventional farmers in Nimar valley did not differ for these characteristics. Average age of the farm head came out to be 44 years for conventional farmers and 45 years for organic farmers. The oldest conventional respondent was 75 years old and youngest was 24 years old whereas among the organic farmers, oldest respondent was 70 years old and youngest respondent was 27 years old.

**Table 1:** Profile of surveyed respondents for gender, age, education, farming experience and land holding.

Farming practices	Conventional				Organic			
	Small	Medium	Large	Overall	Small	Medium	Large	Overall
Male %	100.0	89.3	77.8	88.3	90.9	94.3	64.3	86.7
Female %	0.0	10.7	22.2	13.2	9.1	5.7	35.7	13.3
Average age (Years)	47.6	40.9	44.2	44.2	44.5	46.8	44.9	45.4
Education < 5 year (%)	71.4	71.4	38.9	61.7	72.7	60.0	28.6	55.0
Education > 5 year (%)	28.6	28.6	61.1	38.3	27.3	40.0	71.4	45.0
Experience in farming (Years)	17.8	23.4	28.3	23.2	23.2	21.7	23.8	22.9
Average land holding (ha)	1.25	2.91	7.60	3.9	1.30	2.77	5.46	3.2

**FIGURE 1:**
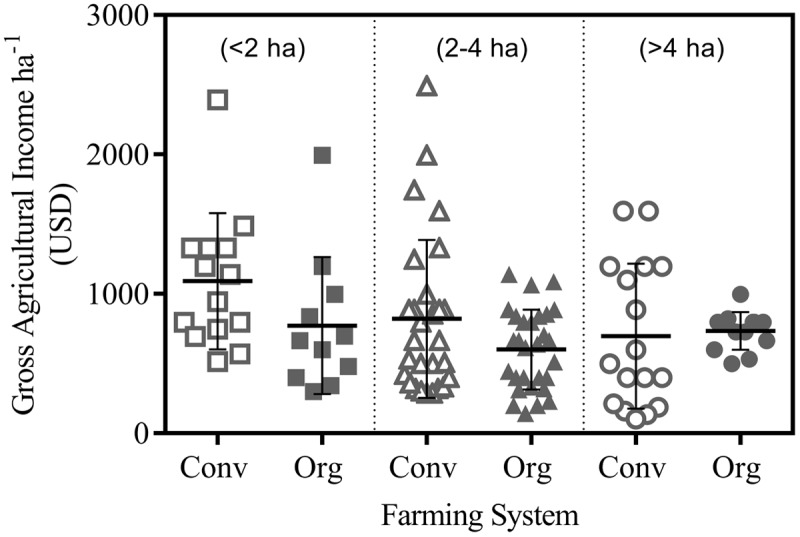
Gross agricultural income ha^-1^ of organic (Org) and conventional (Conv) farmers (1 USD = 62 INR).

Survey also showed that education was low in Small and Medium land holding farmers in both conventional and organic farms. On an average, only 38.3% of conventional farmers and 45.0% of organic farmers had more than 5 years of formal education. Level of education showed positive relationship with the land holding as within large land holding farmers, 61.1% of conventional farmers and 71.4% of organic farmers had more than 5 year of formal education. All surveyed farmers showed similar level of experience in farming (average 23 years; range 18–28 years). Reported gross agricultural income ha^-1^ was comparable across farm sizes and farming systems (**Figure 1**). On conventional farms, median income per unit of land decreased as the land holding increased (**Figure 1**), whereas level of income per unit of land remained unrelated to landholding of organic farmers and did not vary much among the farm sizes.

### Farmers’ View on Major Limiting Factors of Cotton Production in Nimar Valley

**FIGURE 2:**
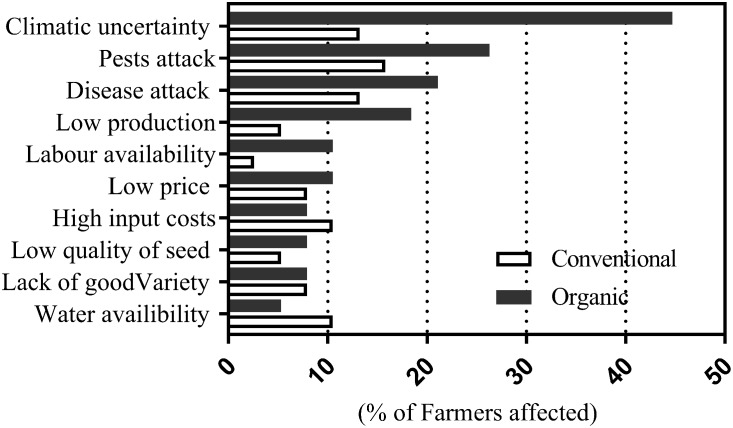
Organic and conventional farmers’ views on major limiting factors of cotton production in Nimar valley.

In an open ended question, conventional and organic farmers were asked about their major concerns on cotton production in Nimar valley. Climatic uncertainty, pest and disease attack were the main concerns of conventional and organic farmers (**Figure 2**). The concerns about climatic uncertainty were raised by proportionately higher number of organic farmers compared to conventional farmers. We found that organic farmers had limited options and capacities for production of botanical extracts to deal with pest and disease incidences. Since seasonal variations have a high degree of influence on frequency and magnitude of pest and disease attacks, the concerns of organic farmers regarding climatic uncertainty indirectly relate to pest and disease attack. Similar concern were also observed in United States by Organic Trade Association (Organic Trade Association, 2015). The conventional farmers interpreted climatic uncertainty in terms of rainfall pattern and distribution throughout the cotton growing season. Low production was the other main concern raised by organic farmers in direct open ended questions, however, elaborated data analysis showed that median yield and yield variation was similar between organic and conventional farms (**Figure 2**). This also shed light on the assumption of satisfactory yield levels, i.e., different farmers could perceive same yield levels as being ‘high’ or ‘low’ depending upon their perspective and awareness. Nevertheless, it is also evident that competitive performance of different agriculture systems vary in different environments and crops; Birkhofer et al. (2008) found that organic system yield 23% lower than conventional system whereas (Reganold et al., 2001) found that organic and conventional system perform similar in apple production.

Labor availability was also a major concern amongst organic farmers compared to conventional. The local farmers perceived that mechanical operations can only be performed on conventional farms whereas organic farming has to be done in more traditional ways. More labor requirement in organic was mainly associated to hand weeding and spraying of the botanical extracts. Lakhal et al. (2008) noted that the organic cotton farmers use 10 times more hired labor than the conventional cotton farmers. Noticeably, the concerns about low price, high input costs, poor quality seed (Hillocks and Kibani, 2002; Page and Ritchie, 2009), lack of high yielding varieties (Page and Ritchie, 2009), and non-availability of water were similar in both organic and conventional farmers.

### Cotton Yield

**FIGURE 3:**
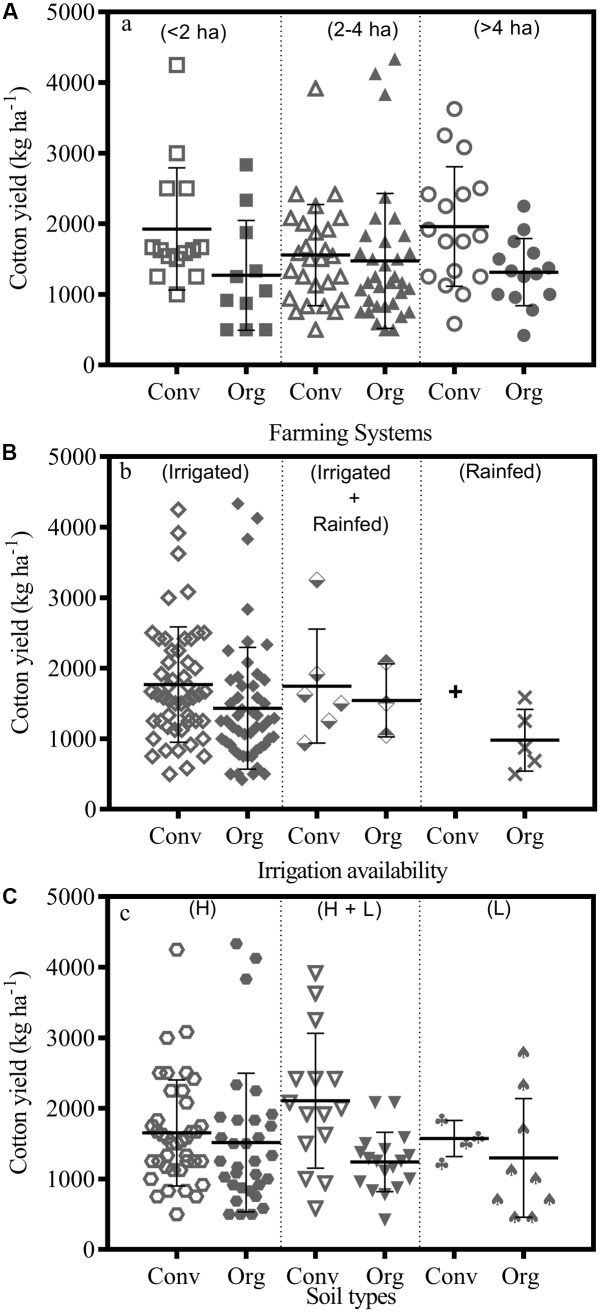
Cotton yield of conventional (Conv) – open circles – and organic farms (Org) – closed circles – in relation to **(A)** farm size, **(B)** irrigation availability, and **(C)** soil types, where each circles represent the reported averaged cotton yield for last 3 years; H: Heavy cotton soil (Vertisol); L: Light soil (Inceptisols and entisols).

A number of factors could influence yield of cotton, crop management practices being the prominent one. Farm size could be a major factor influencing the decision-making and effective implementation of adequate management, whereas irrigation facilities and soil type could be limiting factors for water and nutrient supply to the cotton crop. Farmers were asked to report cotton yield in last 3 years (2012, 2013, and 2014). Means of the reported yields were analyzed against the above-mentioned limiting factors to understand the cotton productivity scenario for both organic and conventional farms in Nimar valley. Analysis showed that the influence of farm size on cotton yield in general was statistically insignificant (**Figure 3A**). The average yield of cotton crop was 1270 ± 383 kg ha^-1^ and 1926 ± 515 kg ha^-1^ on small organic and conventional farms, respectively. Medium sized organic and conventional farms showed comparable cotton yields (1473 ± 253 kg ha^-1^ and 1556 ± 299 kg ha^-1^) with very little variability among the farms. Yield on large size organic farms was 1315 ± 351 kg ha^-1^ compared to 1961 ± 476 kg ha^-1^ on conventional large size farms but both groups did not differ significantly to each other. Most of the surveyed cotton farms had irrigation facilities (**Figure 3B**). The median yield of irrigated cotton organic farms was 1430 ± 121 kg ha^-1^ compared to 1768 ± 115 kg ha^-1^ for irrigated conventional farms. Organic farms with two soil types had lower yield (1239 ± 99 kg ha^-1^) compared to conventional farms that have fields with both soil types (2107 ± 247 kg ha^-1^) (**Figure 3C**). All other groups based on different soil types showed similar yield levels.

Findings from the long-term farming systems comparison experiment located in the same region as this study (Nimar valley) showed that cotton yield in organic production system matched those of conventional production system as soon as the conversion period was over Forster et al. (2013). A farm survey conducted in the same region also showed comparable cotton yields of organic and conventional farms (1459 ± 83 kg ha^-1^ vs. 1400 ± 67 kg ha^-1^) in 2003 and (1237 ± 105 kg ha^-1^ vs. 1166 ± 70 kg ha^-1^) 2004, respectively (Eyhorn et al., 2007). Similarly, in a recent farms survey comparable yields of wheat were found on organic and conventional farms (Helfenstein et al., 2016). In our study, the analysis of three key factors (farm size, irrigation facilities, and soil type) showed that the range of variation among the farms was far-flung, hence it could be concluded that cotton yield gets limited by other factors before it comes to the level where it can be limited primarily by water and soil nutrients. In each category, there were some farms with relatively high productivity as well as with poor productivity. Widespread variation in cotton yield among the farms also indicates that the first step to increase yield would be to improve management practices of cotton crop at individual farm. Therefore, farmers’ knowledge need to be strengthened to improve their understanding and skills (Misiko et al., 2011).

### Farmers’ Motivational Characters behind Farming Practices

**FIGURE 4:**
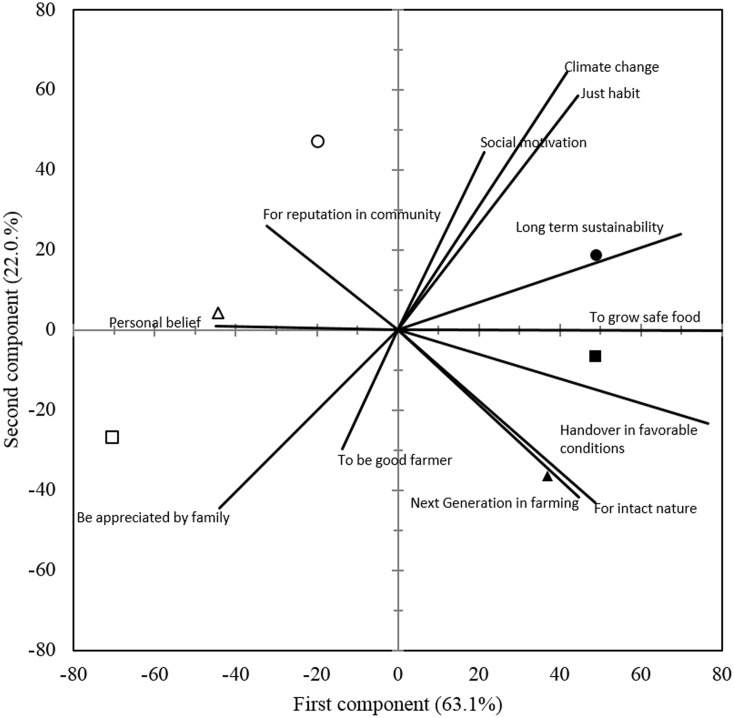
Biplot for the principal component analysis of the respective social motivational characters of (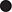) large, (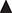) medium, and (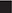) small holding organic farmers; as well as (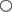) large, (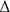) medium, and (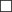) small holding conventional farmers. Closeness of a farming system symbol to a particular motivational character confers the dominance of that motivation, whereas length of the vector line signifies the effect of that motivational character.

**FIGURE 5:**
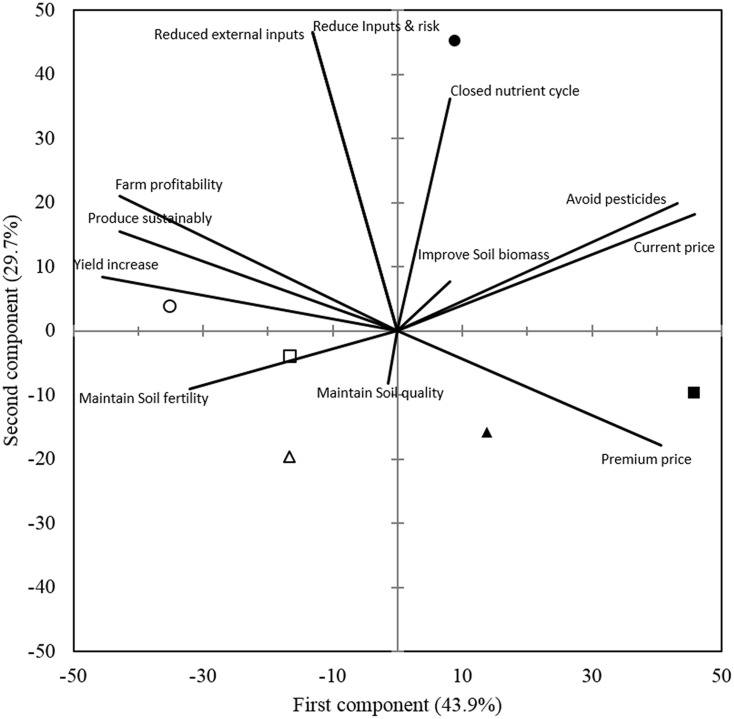
Biplot from the principal component analysis of the biophysical factors influencing the adoption of specific practices by (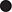) large, (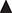) medium, and (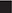) small holding organic farmers as well as (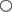) large, (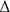) medium, and (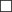) small holding conventional farmers.

While there are no differences among organic and conventional farmers with regard to their age, education, experience and farm size, there must be some other factors influencing their decision to choose either organic or conventional way of farming. We used principle component analysis (PCA) to identify the social, economic, and biophysical motivations of different farmers for following their respective farming practices. PCA provided an overview of the relationship of organic and conventional farming practices on different sized farms to social motivational characters of the farmers as well as to the biophysical reasons perceived by them (**Figures 4**, **5**). In the biplot figures (**Figures 4**, **5**), the axis labels indicate the extent to which the mentioned factors account for the total variation in data. The proximity of a farming system group to a particular motivational character demonstrates the agreement of the farmers in that group to the influence from that character and the length of the vector shows the degree of influence compared to other characters.

### Social Motivational Characters

Analysis of survey data revealed that the motivational characters vary among farmers following specific farming practice and having different farm sizes. Besides the differences among different farm sizes, the points pertaining to organic and conventional farm groups spread into different coordinate quadrants (**Figure 4**) indicate the ideological differences among the followers of these two production systems. The first component of PCA accounting for 63.1% of the total variation, and first component + second component accounting for 85.1% of the total variation showed that these are the most common listed social motivational factors that impact on adoption of a specific management system for cotton production. Some of the social motivation factors such as perception of climate change, habitual reasons, long-term sustainability, interest to grow safer food and societal influence were more important on total variation than others as indicated by the long length of vectors in **Figure 4**. Studies conducted in Canada and United States have reported similar concerns as motivation of farmers for converting to organic, e.g., concerns over environmental impact of farming (Henning, 1994) and motivation for personal, family, or consumer health and safety (Cacek and Langner, 1986; Lockeretz and Madden, 1987; Molder et al., 1991; Henning, 1994; Hall and Mogyorody, 2001; Cranfield et al., 2010).

Long-term sustainability of cotton was the major motivation for organic farmers with larger land holdings (>4 ha). Whereas, growing safer food without pesticides and a wish to handover their land to the next generation in a better condition were expressed as main motivations by the organic farmers with medium sized holdings (2–4 ha). However, it is noteworthy that only 32.3% of the surveyed organic medium holding farmers wanted their children to become farmer 1 day. Motivation of small holding (<2 ha) organic farmers was to perform agricultural practices that are favorable for an intact nature and 33.3% of them wanted their children to become farmers 1 day. In contrary to organic farmers, the motivation of conventional farmers was ambiguous. Large holding conventional farmers did not seem to derive their motivation from the mentioned social factors as indicated by the remote presence of point pertaining to this group in 2nd quadrate (**Figure 4**). The closest vector indicated that they were only concerned about their reputation in the community. Medium holding conventional farmers believed that the conventional practice was a better way of farming (personal belief). However, the small holding conventional farmers seemed to be aloof of the studied social factors and therefore, the social motivation of this farming group remains unclear. The closeness to vectors of ‘personal belief’ and ‘appreciation from family’ may suggest lack of awareness and limited risk bearing ability, preventing a shift from the existing farming practices.

### Biophysical and Economic Motivational Characters

Similar to the social motivational characters, the points pertaining to organic and conventional farming groups with different farm holdings were spread into different coordinate quadrants, clearly distinguishing the biophysical motivational characters of each group. As the first and second component together account for 73.6% of the total variation, it means that the listed biophysical factors are the most common ones influencing the surveyed organic and conventional farms (**Figure 5**). Current price of cotton, avoiding the exposure to pesticides and closed nutrient cycles turned out to be more important factors on total variation than other ones, as indicated by their long length of vectors in the first quadrant. Reduction of the production costs and risk of ineptness by being independent of external inputs as well as the premium price were some other important factors for organic farmers. Closer review of the responses revealed that large holding organic farmers were more concerned about closed nutrient cycles to reduce their dependence on external inputs, whereas medium and small holding organic farmers were clearly motivated by the premium price of organic cotton. Results of this study as well as previously conducted studies in advanced economies reveal that profitability/financial return is gaining importance as a stronger decision-making factor in opting for organic. In a survey conducted by Henning (1994), only 9% of the study respondents indicted profitability as important factor, whereas in a survey of 2001, 56% of the respondents mentioned profitability as *very important* factor for conversion to organic agriculture (Hall and Mogyorody, 2001). On the other hand large holding conventional farmers in our study did not opt for organic agriculture as they believed that high yield was the key to success which could only be achieved by conventional practices. As in the case of social motivational factors, medium holding conventional farmers did not have any clear consideration of biophysical factors for adoption of conventional farming. Small holding conventional farmers believed that the application of fertilizers is important to improve the fertility of their soils. In addition, opportunistic decisions influenced by changed circumstances could contribute to farmers’ adoption or abandoning of a specific farming system. For instance, Eyhorn et al. (2007) reported 30–40% fallback rate of organic cotton farmers to conventional practices under the influence of campaign by companies selling newly introduced Bt-cotton seed in 2003.

### Preference to Grow Organic Cotton

Apart from the PCA comparing different farming groups, we also sought to find out the relative importance of different factors considered important by organic farmers for adoption of organic practices. Low production cost followed by premium price, cash payment and door-step purchasing were the main motivating factors to grow organic cotton in west Nimar valley (**Table 2**). Farmers’ responses explained that financial motivation was the main driving factor for the cotton production followed by sustainability (soil health + stable production) and hassle free management of organic cotton crop. Rigby and Cáceres (2001) also identified the financial motivation and soil health as two out of four major key motivational factors for organic farming in United Kingdom.

**Table 2:** Main reasons of farmers to opt for organic cotton production and the proportion of farmers assigning importance to each.

Response	% farmers
Low production cost	31.6
Premium price	16.5
Cash payment	12.0
Door-step purchasing	12.0
Improves the soil health	12.0
Stable production	3.8
No wilting problem	3.0
Easy seed availability from contractor	2.3
Personal preference	2.3
Intact nature	1.5
No disease	1.5
No dependency on market	0.8
Low risk	0.8

### Switching from Conventional to Organic

In contrary to organic farmers, the conventional farmers were asked about the potential circumstances under which they can switch from conventional to organic farming. Surprisingly only six key responses surfaced, which clearly showed that conventional farmers were very clear in making the comparisons about the ground situation of organic and conventional farming (**Table 3**). Cranfield et al. (2010) reviewed the literature and categorized the motivational factors for conversion into four broad themes of (a) financial issues; (b) environmental concerns; (c) philosophical motives; and (d) health and safety concerns. Out of six key potential circumstances of cotton grower for conversion four fell into first three themes [response 1, 2, 5, and 6 (**Table 3**)]. However, in-depth analysis of motivational factor revealed that even health and safety concerns are not untouched in this part of the world and remained a subconscious motivation of organic cotton growers in Nimar valley (**Figure 5**). Similar to organic farmers, main motivation of the conventional farmers for potential conversion was also to achieve economic profit either by high yield and high price, low input cost or by hassle free management (**Table 3**).

**Table 3:** Scenarios for shifting from conventional to organic farming practices.

Sr. No.	Response	% farmers
1	When cost benefit ratio will further decline due to high input costs related to conventional farming practices	61.9
2	When soil fertility become too low, I would opt for organic agriculture to maintain it.	23.8
3	If I get substantial support from private/govt. sector for organic	6.3
4	If there is no solution of Bt-cotton wilting	4.8
5	Higher premium price	1.6
6	When more farmer of my region will opt for organic agriculture I also will go for organic.	1.6

## Policy Implications of Inferences

The findings of this study confirm our hypothesis that the motivational characteristics of farmers for adoption of conventional or organic farming systems differs depending upon their awareness level, social perceptions, availability of resources and perceived profitability. In addition, the study results provide a detailed diagnoses of the biophysical and socio-economic factors influencing the rationale behind decision of the cotton farmers to adopt organic or conventional production systems. The inferences from this study could contribute toward the development and implementation of suitable policies promoting organic/sustainable farming systems. For instance, the large variation among cotton yields achieved by both the organic and conventional farmers highlights the tremendous scope of improvement of cotton productivity. If the underperforming farms are supported to increase their production, even to the average levels, significant increase in overall production could be achieved. In some cases, the farmers (particularly the small holders) are not even aware of the potential of increasing yields by available technologies. This is an important open area to be addressed by extension and policy institutions in collaboration with research. Innovation platforms aimed at local capacity building and development of locally adapted technologies could serve as an important tool in this direction (Andres et al., 2016).

Social motivational factors vary among organic and conventional farmers, as organic farmers are motivated by the sustainability of cotton production, growing safer food without pesticides and a wish to hand over their land to their successors in favorable condition, while the major motivation of conventional farmers is their reputation in community. Considering this, incentivising the sustainable farms for ecosystem services they provide would be an important policy measure toward achieving sustainability in agricultural systems. In case of the biophysical factors, organic farmers with larger holdings are more concerned about closed nutrient cycles and reducing their dependence on external inputs, whereas medium and small holding organic farmers are clearly motivated by the premium price of organic cotton. Since 80% farmers in India are small and medium holder, financial support during the conversion period from conventional to organic production system could serve as important driver of change to bring them on board. Higher productivity is the only important motivation for conventional farmers with larger land holdings. These results suggest that it is important to close the knowledge gap by strengthening extension services. Simultaneous and continuous training of extension workers and farmers in sustainable farming practices is of high value and thus deserves due diligence. It is also important that the farmers are made aware of the scope of increasing yields and the potential of existing technologies. Creating the awareness about yield gap and yield variation among the farmers and encouraging them to achieve maximum attainable yield by using the examples of high yielding farms could be a useful approach. Efforts need to be directed at improving the timely availability of quality on-farm inputs for organic production such as seeds and pest control measures. Moreover, research efforts need to be intensified to make available locally developed technologies and improved organic practices for nutrition, plant protection as well as agronomic management. Providing suitable marketing opportunities by developing value chains for organic produce other than cash crops (organic cotton in this case) will also be important to maintain the motivation and commitment of organic farmers as well as will provide level economic ground.

## Ethics Statement

This study was carried out in accordance with the internationally accepted ethical standards for social studies and was approved by the ‘Farmer Advisory committee’ of bioRe Association, India. All subjects (interviewed farmers) gave written and informed consent. A formal ethics approval for this study was not needed as per our Institutional guidelines and per the relevant Indian regulations and laws.

## Author Contributions

AR and GB designed the study; LM and AR coordinated the data collection; AR analyzed the data; AR and GB prepared the first draft of manuscript; MM and RP supported the design and analysis; all authors revised the manuscript.

## Conflict of Interest Statement

The authors declare that the research was conducted in the absence of any commercial or financial relationships that could be construed as a potential conflict of interest.
